# Using Linkage to Electronic Primary Care Records to Evaluate Recruitment and Nonresponse Bias in The Avon Longitudinal Study of Parents and Children

**DOI:** 10.1097/EDE.0000000000000288

**Published:** 2015-06-02

**Authors:** Rosie Cornish, Kate Tilling, Andy Boyd, John Macleod, Tjeerd Van Staa

**Affiliations:** School of Social and Community Medicine, University of Bristol, Bristol, United Kingdom, rosie.cornish@bristol.ac.uk; School of Social and Community Medicine, University of Bristol, Bristol, United Kingdom; MRC Integrative Epidemiology Unit, School of Social and Community Medicine, University of Bristol, Bristol, United Kingdom; School of Social and Community Medicine, University of Bristol, Bristol, United Kingdom; Health e-Research Centre, University of Manchester, Manchester, United Kingdom; Utrecht Institute for Pharmaceutical Sciences, Utrecht University, Utrecht, The Netherlands

## Abstract

Supplemental Digital Content is available in the text.

## To the Editor:

Participation is often incomplete in observational research—because of initial failure to recruit or loss to follow-up—resulting in loss of statistical power and possible bias. Selective participation often leads to biased prevalence estimates, but appears to be less important in relation to estimates of exposure–outcome associations, though exceptions have been reported.^1–7^

Linkage to routine health data offers a means to examine the extent of these biases by providing data on participants and nonparticipants in a study. Here, within appropriate permissions, we have linked those eligible to take part in The Avon Longitudinal Study of Parents and Children (ALSPAC), a birth cohort, to the General Practice Research Database, an anonymized database of primary care records of around 5 million UK residents. The linked sample comprised 749 individuals eligible to take part in ALSPAC (4% of the ALSPAC-eligible population). Of these, 519 (69%) had enrolled (originally been recruited) in ALSPAC, of whom 348 (67%) had participated at 10 years of age and 223 (43%) at 17 years of age. Further details about the sample are given in the eAppendix (http://links.lww.com/EDE/A898).

We used the linked resource to define the cumulative incidence (by ages 11 and 19) of six outcomes: any mental illness, any respiratory illness (excluding asthma), asthma and/or allergies, ever smoked (up to 19 only), been pregnant (19 only), and been classified as “child at risk” (eAppendix; http://links.lww.com/EDE/A898). Data on exposures and potential confounders were obtained from ALSPAC questionnaires administered during pregnancy and early infancy.

For each outcome, we calculated the cumulative incidence among those eligible to take part in ALSPAC, those who had enrolled and those who participated at 10 and 17 years of age. We compared these using ratios of cumulative incidences. We calculated odds ratios (ORs) for four exposure–outcome associations (three examined separately at 10 and 17 years of age) among all enrolled subjects, and compared them—using relative odds ratios (RORs)—to ORs from individuals participating at 10 and 17 years of age. Confidence intervals were constructed around the logarithm of the ratios of cumulative incidence and RORs using a nonparametric bootstrap method.

The cumulative incidence of each of the outcomes was similar among enrolled and eligible subjects (eTable 4; http://links.lww.com/EDE/A898). However, participants were less likely than all enrolled subjects to have ever smoked, been pregnant or been “at risk” (eTable 5; http://links.lww.com/EDE/A898). For all the exposure–outcome associations, the ORs among participants at 17 were substantially different from those among all enrolled subjects (Table), although confidence intervals were inevitably wide given the small numbers with linked data available. The RORs for participants at 10 years of age were all closer to unity. Adjusting for other factors predictive of nonparticipation made little difference to these RORs. For three of the outcomes, there was evidence for an interaction between the exposure and outcome with respect to participation at 17 years of age, resulting in the outcome being missing not at random in one exposure group but not the other (eTable 6; http://links.lww.com/EDE/A898). These interactions were not seen at 10 years of age.

**TABLE.**
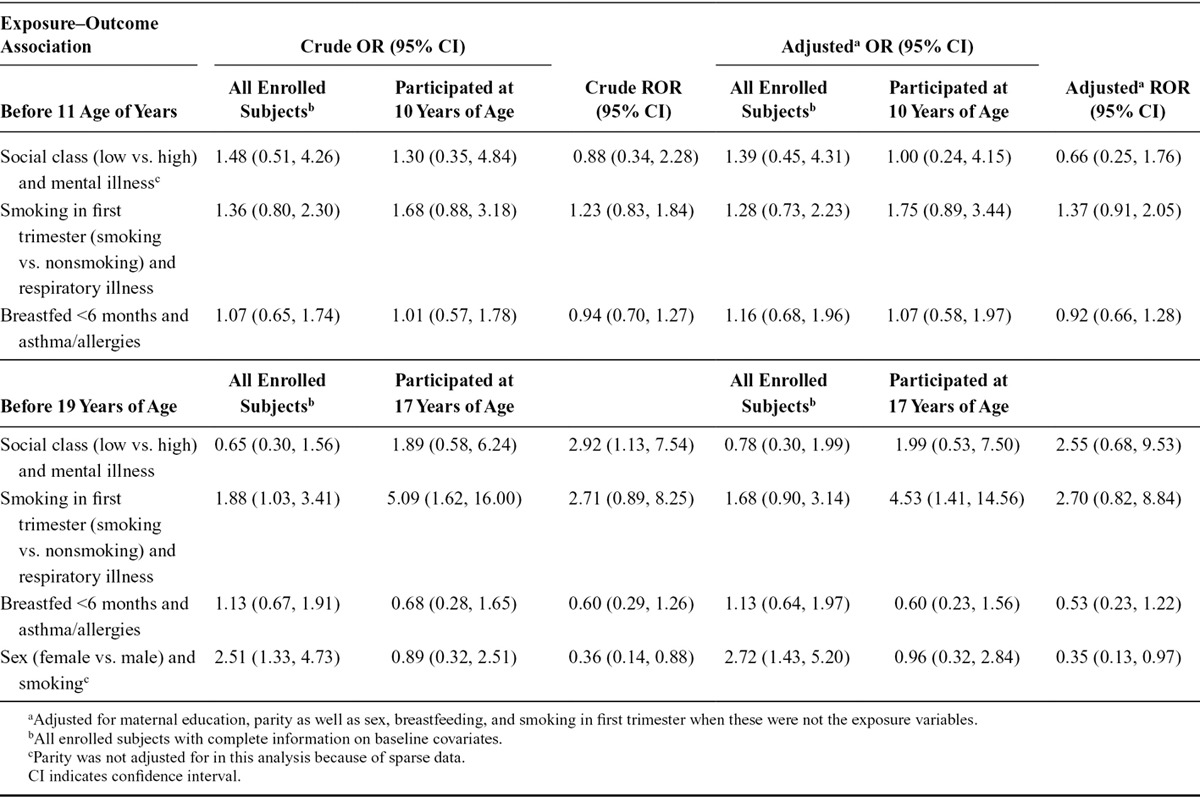
Crude and Adjusted Relative Odds Ratios (RORs) for Different Exposure–Outcome Associations Measured up to 11 Years and up to 19 Years Among Subjects Enrolled in the Avon Longitudinal Study of Parents and Children Also Appearing in the General Practice Research Database

In conclusion, we have shown that bias due to loss to follow-up in some exposure–outcome associations may be substantial, although this study was small and the uncertainty in our estimates of bias consequently quite large. Our study demonstrates that linkage between observational studies and electronic patient records can provide useful information on nonresponders. This utility is likely to increase as the proportion of individuals with linked data increases. Comparative information available via linkage could be combined with, or used in place of, observational data. In addition, linked data could provide important information on factors predictive of nonresponse; such information can be incorporated into statistical analyses, thus reducing bias^8^ and potentially increasing the precision of estimates.

## ACKNOWLEDGMENTS

We are extremely grateful to all the families that took part in this study, the midwives for their help in recruiting them, and the whole ALSPAC team, which includes interviewers, computer and laboratory technicians, clerical workers, research scientists, volunteers, managers, receptionists, and nurses.

## Supplementary Material

**Rosie Cornish** School of Social and Community Medicine University of Bristol Bristol, United Kingdom rosie.cornish@bristol.ac.uk**Kate Tilling** School of Social and Community Medicine University of Bristol Bristol, United Kingdom MRC Integrative Epidemiology Unit School of Social and Community Medicine University of Bristol Bristol, United Kingdom**Andy Boyd** **John Macleod** School of Social and Community Medicine University of Bristol Bristol, United Kingdom**Tjeerd Van Staa** Health e-Research Centre University of Manchester Manchester, United Kingdom Utrecht Institute for Pharmaceutical Sciences Utrecht University Utrecht, The Netherlands

## References

[1] Wolke D, Waylen A, Samara M (2009). Selective drop-out in longitudinal studies and non-biased prediction of behaviour disorders.. Br J Psychiatry.

[2] Howe LD, Tilling K, Galobardes B, Lawlor DA. (2013). Loss to follow-up in cohort studies: bias in estimates of socioeconomic inequalities.. Epidemiology.

[3] Ferrie JE, Kivimäki M, Singh-Manoux A (2009). Non-response to baseline, non-response to follow-up and mortality in the Whitehall II cohort.. Int J Epidemiol.

[4] Greene N, Greenland S, Olsen J, Nohr EA. (2011). Estimating bias from loss to follow-up in the Danish National Birth Cohort.. Epidemiology.

[5] Bjertness E, Sagatun A, Green K, Lien L, Søgaard AJ, Selmer R. (2010). Response rates and selection problems, with emphasis on mental health variables and DNA sampling, in large population-based, cross-sectional and longitudinal studies of adolescents in Norway.. BMC Public Health.

[6] Tin Tin S, Woodward A, Ameratunga S. (2014). Estimating bias from loss to follow-up in a prospective cohort study of bicycle crash injuries.. Inj Prev.

[7] Osler M, Kriegbaum M, Christensen U, Lund R, Nybo Andersen AM. (2008). Loss to follow up did not bias associations between early life factors and adult depression.. J Clin Epidemiol.

[8] Gorman E, Leyland AH, McCartney G (2014). Assessing the representativeness of population-sampled health surveys through linkage to administrative data on alcohol-related outcomes.. Am J Epidemiol.

