# Supplementary material for: Using Linkage to Electronic Primary Care Records to Evaluate Recruitment and Nonresponse Bias in The Avon Longitudinal Study of Parents and Children
Source: Epidemiology. 2015 Jun 2;26(4):e41–2. doi: 10.1097/EDE.0000000000000288 (PMC4454477; doi:10.1097/EDE.0000000000000288)
Supplement: Supplementary file 1 [file ede-26-e41-s001.docx]

**Using linkage to electronic primary care records to evaluate recruitment and non-response bias in the Avon Longitudinal Study of Parents and Children: eAppendix**

This appendix contains additional information about the methods and results and a detailed discussion.

**Methods**

Subjects

Subjects were those eligible to participate in ALSPAC who also had a record in the GPRD, which has now become part of the Clinical Practice Research Datalink [<http://www.cprd.com>]. The GPRD is an anonymised database of primary care records of around 5 million patients in the UK, covering approximately 8% of the UK population; practices using Vision software (this replaced VAMP Medical software and is one of several software systems used by GP practices in the UK) contribute to the database. Patients are part of the GPRD from the time at which they register into a practice that contributes to the database; similarly they leave the GPRD if they transfer to a practice that does not contribute. Thus, follow-up in GPRD is not complete for all individuals. ALSPAC has been described in detail before.^1^ To summarise, 20248 pregnant women living in and around Bristol, UK with due dates between 1 April 1991 and 31 December 1992 were eligible to take part in the study. This eligible population was determined retrospectively using study recruitment records as well as maternity, birth and child health records. Because there was no sampling frame to enable systematic recruitment of all eligible individuals, ALSPAC recruitment was opportunistic and done through antenatal services. Of the eligible women, 14541 enrolled in the study in 1990-1992 and a further 706 in later years. These enrolled pregnancies resulted in 14775 live-born children, of which 14701 were alive at one year. The enrolled participants have been followed up regularly since birth. Further details of the study are given on ALSPAC’s website (<http://www.bristol.ac.uk/alspac>), which also includes a fully searchable data-dictionary of all available data (<http://www.bris.ac.uk/alspac/researchers/data-access/data-dictionary>. Attrition rates in ALSPAC were highest in infancy and late adolescence and just over 5000 participants completed the questionnaire sent at approximately 16½ years (response rates have been described in detail elsewhere).^1^ Previous analysis using data from ALSPAC has shown that those who participated in the study between 16 and 18 years were more likely to be female, more likely to be white and less likely to live in low income households.^1^ However, little is known about how the subjects who enrolled in ALSPAC differ from those who were eligible to take part in the study but did not enrol. Ethical approval was obtained from the ALSPAC Ethics and Law Committee, Local Research Ethics Committees and the NHS National Information Governance Board (NIGB).

Linkage between ALSPAC and the GPRD

Linkage between ALSPAC and the GPRD was conducted by the NHS Information Centre (NHS IC) in the role of a trusted third party and using a method to preserve anonymity. The NHS IC had ascertained the NHS numbers of individuals meeting recruitment criteria and eligible to participate in ALSPAC – regardless of whether or not they enrolled in the study – as part of a previous linkage exercise.^1^ With approval from the NIGB Ethics and Confidentiality Committee, the NHS IC used this information to identify ALSPAC-eligible individuals who also appeared in the GPRD; they then sent an anonymised linking dataset to be stored securely at the GPRD. ALSPAC and GPRD data for linked individuals were merged and analysed in a safe setting at the GPRD offices. As the GPRD is anonymous and collected on an opt-out basis, and anonymity was preserved using the safeguards described above, this piece of research does not require consent above and beyond the consent obtained for participation in ALSPAC and inclusion in GPRD. However, ALSPAC has been collecting consent from participants, who are now adults, for ongoing participation in the study as well as consent to extract information from health and other administrative records and any individuals who withdrew from the study or did not agree to their health records being extracted were excluded from the linkage.

GPRD measures (outcome variables)

The definition of being “child at risk” was: an individual ever having a Read code (the clinical coding system used in primary care in the UK) in their record indicating they were at risk of abuse or neglect; these codes were based on the RCGP/NSPCC (Royal College of General Practitioners/National Society for the Prevention of Cruelty to Children) Safeguarding Children Toolkit.^2^ The Johns Hopkins University Adjusted Clinical Groups (ACG®) System (<http://acg.jhsph.org/>), which was used to construct three of the outcome measures, considers all the relevant Read codes from a person’s record and categorises these as one of 267expanded diagnostic clusters (EDCs), a classification of clinically similar conditions. These EDCs are grouped into 27 categories, depending on the type of illness. For example, cardiovascular conditions, renal conditions, and so on. The groupings used in this study contain the following EDCs: mental illness – anxiety/neuroses, substance use, behaviour problems, attention deficit disorder, family and social problems, schizophrenia and affective psychosis, personality disorders, depression, bipolar disorder, psychosocial disorders (other), and psychologic signs and symptoms; respiratory illness – acute lower respiratory tract infection, cystic fibrosis, emphysema, chronic bronchitis, COPD, cough, sleep apnea, sinusitis, pulmonary embolism, tracheostomy, respiratory failure, respiratory disorders (other), respiratory signs and symptoms; and asthma/allergies – asthma, allergic reactions, allergic rhinitis. These outcomes are very broad; however, the aim was not to accurately quantify the effect of particular exposures on the outcomes being considered, but rather to illustrate the potential extent of non-response bias.

For all of the outcome measures, subjects who left a GPRD-contributing practice before the applicable age cut-off (11 years or 19 years) and, up to the point of them leaving the GPRD, did not have a positive diagnosis of the outcome being considered were excluded from the analysis for that outcome as it could not be determined whether or not these events occurred subsequently (i.e. between them leaving the GPRD and 11 or 19 years).

ALSPAC measures (exposures and potential confounding factors)

Mothers were asked at 18 weeks gestation whether they had smoked during the past 3 months. Maternal and paternal educational status and maternal and paternal occupational social class were also determined during pregnancy. Family occupational social class was defined as the higher of paternal and maternal social class and was classified as manual (social classes IIIM-IV) or non-manual (IIIN-I). The breastfeeding information used for this analysis was collected at 6 months; subjects were classified according to whether or not they were ever breastfed in the first 6 months.

Participation in ALSPAC at 10 years was defined as having attended the clinic scheduled when the children were 10 years old or completed a questionnaire (either child-completed or parent/carer-completed) administered between the ages of 9½
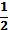
 and 11½
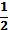
 years; similarly, participation at 17 years was defined as having completed an ALSPAC assessment during the “transition to adulthood” phase (either completed a questionnaire administered at age 16 years, a questionnaire at age 18, or attended the clinic scheduled at 17 years).

Summary of terms used

**Eligible:** met the ALSPAC recruitment criteria (living in and around Bristol) and with due dates between 1^st^ April 1991 and 31^st^ December 1992. The eligible group was determined retrospectively from ALSPAC records as well as maternity, birth and child health records.

**Enrolled:** the mother enrolled into the study.

**Participated:** we defined participation at two ages - 10 and 17 – as described above.

**Results**

Of all the (ALSPAC-eligible) live births linked by the NHS IC, 765 appeared in the GPRD; fifteen of these declined consent for linkage to their health records and one registered into and left GPRD on the same day, resulting in no follow up. Of the remaining 749 eligible individuals, 519 (69%) had enrolled in ALSPAC. This percentage is slightly lower than the percentage of all ALSPAC-eligible children alive at one year who enrolled into the study – 14701/19498 (75%). Among those enrolled, 348 (67%) had participated in ALSPAC at around 10 years, and 223 (43%) had participated at 17 years (see eFigure 1). These percentages reflect similar losses in the ALSPAC-enrolled cohort. Of the 519 enrolled subjects, 365 had complete baseline data. Most of this loss was due to missing data on breastfeeding as this information was collected at 6 months rather than during pregnancy (among the ALSPAC-enrolled cohort, only 11490 individuals filled in the 6-month questionnaire).

eFigure 1: Flowchart of ALSPAC subject participation in relation to presence in the GPRD

ALSPAC cohort ALSPAC individuals

within the GPRD

All pregnant women living in the county of Avon with due dates between April 1991 & December 1992 were eligible to take part in ALSPAC (n=20,248 pregnancies, 19,498 children alive at one year).

765 ALSPAC-eligible subjects had a record in the GPRD; 749 did not dissent to linkage and had ≥1 day of GPRD follow up. These 749 subjects comprised the eligible group in our analysis (3.8% of the original eligible children alive at one year).

749

14,701 children were enrolled in ALSPAC and were alive at one year.

519 of those eligible had enrolled in ALSPAC (3.5% of enrolled children alive at one year); 365 of these had complete baseline data on parity, maternal education, breastfeeding and smoking in pregnancy.

348 participated at around 10 years. (271 with complete baseline data).

223 participated at around 17 years (180 with complete baseline data).

The original enrolled ALSPAC sample were similar to the 519 enrolled individuals who were also in the GPRD with respect to parity, family occupational social class, and paternal education; however, there were differences in the distribution of maternal age and education. These figures are shown in eTable 1.

| eTable 1: Characteristics of the original ALSPAC enrolled sample compared to the enrolled individuals appearing in the GPRD | | | |
| --- | --- | --- | --- |
|  |  | Original ALSPAC enrolled sample | Enrolled sample appearing in the GPRD |
| Maternal education | O level or lower  A level or higher | 65%  35% | 73%  27% |
| Paternal education | O level or lower  A level or higher | 51%  49% | 50%  50% |
| Occupational social class | I-IIIN  IIIM-IV | 81%  19% | 79%  21% |
| Mother’s age | <20  20-24  25-29  30-34  35+ | 5%  19%  39%  28%  10% | 5%  23%  44%  20%  7% |
| Parity | 0  1  2+ | 45%  35%  20% | 45%  35%  20% |

Basic characteristics of subjects according to participation are shown in eTable 2. Those who enrolled in ALSPAC were similar to those who did not enrol. However, those who had participated at ages 10 and 17 were more likely to be female and come from less deprived areas, as measured by the Index of Multiple Deprivation (IMD) 2007.

| eTable 2: Sex and IMD (2007) of subjects according to enrolment and participation in ALSPAC | | | | | | | |
| --- | --- | --- | --- | --- | --- | --- | --- |
|  | Sex |  | IMD (2007) quintile | |  | |  |
|  | Male | Female | Least deprived | 2^nd^ | 3^rd^ | 4^th^ | Most deprived |
| Enrolled  Eligible but not | 260  118 | 259 (50%)  112 (49%) | 50 (13%)  15 (9%) | 119 (31%)  52 (30%) | 93 (24%)  38 (22%) | 40 (11%)  23 (13%) | 78 (21%)  44 (26%) |
| enrolled | (χ^2^_1_=0.09, p=0.8) | | (χ^2^_4_=4.5, p=0.3) | | | | |
| Participated at 10 Enrolled, but did | 166  94 | 182 (52%)  77 (45%) | 41 (16%)  9 (7%) | 88 (35%)  31 (24%) | 63 (25%)  30 (23%) | 18 (7%)  22 (17%) | 40 (16%)  38 (29%) |
| not participate at 10 | (χ^2^_1_=2.4, p=0.1) | | (χ^2^_4_=24.5, p<0.001) | | | | |
| Participated at 17  Enrolled but did | 92  168 | 131 (59%)  128 (43%) | 30 (18%)  20 (9%) | 55 (33%)  64 (30%) | 47 (28%)  46 (21%) | 10 (7%)  30 (14%) | 23 (14%)  55 (26%) |
| not participate at 17 | (χ^2^_1_=12.5, p<0.001) | | (χ^2^_4_=19.6, p=0.001) | | | | |

eTable 3 shows the distributions of factors measured by ALSPAC during pregnancy according to participation status. Non-participating subjects, both at 10 and 17 years were less likely to have been breast fed and to be in a higher socio-economic group; their mothers were more likely to be aged under 25, to have smoked during pregnancy and have lower educational outcomes. Paternal education was weakly associated with participation; parity was associated with participation at age 17 but not at 10. After mutual adjustment (for sex plus all the factors listed in eTable 3), participation at age 10 remained associated with smoking in pregnancy (OR=0.52, 0.27-1.00, p=0.05) and maternal education (OR=2.64, 1.24-5.59, p=0.01); the factors that remained associated with participation at age 17 were sex (OR=2.20, 95% CI 1.35-3.58, p=0.002), maternal education (OR=1.87, 1.04-3.37, p=0.04), breastfeeding (OR=1.84, 1.03-3.30, p=0.04) and parity (OR=1.50, 0.86-2.64, comparing 1 to 0; OR=0.47, 0.23-0.98 comparing 2+ to 0, p=0.007). Thus, from the data available through ALSPAC, we can conclude that data were not missing completely at random but, if we did not have outcome data obtained via linkage, we would not be able to distinguish between data missing at random (missingness depends only on the observed data) and data missing not at random (missingness depends additionally on the outcome variables).

| eTable 3: Factors associated with participation in ALSPAC^a^, among subjects enrolled in ALSPAC who also appeared in the GPRD | | | | | | | |
| --- | --- | --- | --- | --- | --- | --- | --- |
|  |  | Participated at 10 years | | | Participated at 17 years | | |
|  |  | No | Yes | p-value^b^ | No | Yes | p-value^b^ |
| Maternal education | O level or lower  A level or higher | 129  21 | 205 (61%)  103 (83%) | p<0.001 | 209  51 | 124 (37%)  73 (59%) | p<0.001 |
| Paternal education | O level or lower  A level or higher | 60  48 | 128 (68%)  138 (74%) | p=0.2 | 106  88 | 82 (44%)  97 (52%) | p=0.09 |
| Occupational social class | I-IIIN  IIIM-IV | 94  41 | 232 (71%)  50 (55%) | p=0.003 | 164  62 | 16 (50%)  29 (32%) | p=0.003 |
| Mother’s age | <20  20-24  25-29  30-34  35+ | 16  65  67  33  9 | 12 (43%)  55 (46%)  163 (71%)  73 (69%)  26 (74%) | p<0.001 | 22  86  129  56  20 | 6 (21%)  34 (28%)  100 (44%)  50 (47%)  15 (43%) | p=0.006 |
| Parity | 0  1  2+ | 77  54  37 | 140 (65%)  114 (68%)  62 (63%) | p=0.7 | 118  92  74 | 97 (45%)  76 (45%)  25 (25%) | p=0.002 |
| Ever breast fed in first 6 months | No  Yes | 36  64 | 75 (68%)  209 (77%) | p=0.07 | 72  123 | 38 (35%)  150 (55%) | p<0.001 |
| Smoking in the first trimester | No  Yes | 104  68 | 251 (71%)  69 (50%) | p<0.001 | 188  100 | 165 (47%)  37(27%) | p<0.001 |
| ^a^Denominators in this table vary because all those for whom data were available for a particular variable are included: paternal data and post-pregnancy data are available for fewer subjects.  ^b^From the univariate analysis (chi-squared test) | | | | | | | |

The cumulative incidence of each outcome among all those eligible to take part in ALSPAC and enrolled subjects are given in eTable 4. The outcomes were similar among enrolled and eligible subjects, although the proportions classified as at risk were lower among the former.

| eTable 4: Ratios of cumulative incidence (RCIs) for outcomes measures among all those eligible to participate in ALSPAC and all enrolled subjects appearing in the GPRD | | | | | |
| --- | --- | --- | --- | --- | --- |
| Outcome measure | |  | Eligible subjects | Enrolled subjects | RCI (95% CI) (enrolled/eligible) |
| **Before age 11** |  | |  |  |  |
| At risk | No  Yes | | 624  16 (3%) | 436  8 (2%) | 0.72 (0.44, 1.18) |
| Mental illness | No  Yes | | 598  45 (7%) | 411  34 (8%) | 1.09 (0.92, 1.29) |
| Respiratory illness | No  Yes | | 459  215 (32%) | 312  155 (33%) | 1.04 (0.97, 1.11) |
| Asthma/allergies | No  Yes | | 440  231 (34%) | 297  172 (37%) | 1.07 (1.00, 1.14) |
| **Before age 19** |  | |  |  |  |
| At risk | No  Yes | | 444  29 (6%) | 314  18 (5%) | 0.88 (0.67, 1.17) |
| Mental illness | No  Yes | | 382  111 (23%) | 265  82 (24%) | 1.05 (0.95, 1.16) |
| Respiratory illness | No  Yes | | 265  278 (51%) | 177  205 (54%) | 1.05 (1.00, 1.10) |
| Asthma/allergies before 19 years | No  Yes | | 263  286 (52%) | 184  214 (54%) | 1.03 (0.98, 1.08) |
| Smoked | No  Yes | | 347  135 (28%) | 247  92 (27%) | 0.97 (0.88, 1.07) |
| Pregnancy | No  Yes | | 199  42 (17%) | 144  28 (16%) | 0.93 (0.78, 1.12) |

The cumulative incidence of each of the outcome measures among all subjects enrolled in ALSPAC and subjects who participated at ages 10 and 17 is given in eTable 5. Subjects who participated were less likely to have ever smoked, been pregnant or to have had an “at risk” Read code in their record. The cumulative incidence of the other outcomes was similar amongst those continuing to participate at ages 10 and 17 to that among all enrolled subjects.

| eTable 5: Ratios of cumulative incidence (RCIs) for outcome measures (cumulative incidence) among all enrolled subjects and those participating in ALSPAC at ages 10 and 17 years | | | | | |
| --- | --- | --- | --- | --- | --- |
| Outcome measure | |  | All enrolled subjects | Participated at 10 years | RRF (95% CI)  (particated/enrolled) |
| **Before age 11** |  | |  |  |  |
| At risk | No  Yes | | 436  X^a^ | 297  X^a^ | 0.37 (0.14, 0.99) |
| Mental illness | No  Yes | | 411  34 (8%) | 278  22 (7%) | 0.96 (0.75, 1.23) |
| Respiratory illness | No  Yes | | 312  155 (33%) | 206  107 (34%) | 1.03 (0.95, 1.12) |
| Asthma/allergies | No  Yes | | 297  172 (37%) | 192  124 (39%) | 1.07 (0.99, 1.15) |
| **Before age 19** |  | | All enrolled subjects | Participated at 17 years |  |
| At risk | No  Yes | | 314  X^a^ | 139  X^a^ | 0.39 (0.14, 1.07) |
| Mental illness | No  Yes | | 265  82 (24%) | 109  37 (25%) | 1.07 (0.87, 1.32) |
| Respiratory illness | No  Yes | | 177  205 (54%) | 79  87 (52%) | 0.98 (0.87, 1.10) |
| Asthma/allergies | No  Yes | | 184  214 (54%) | 79  94 (54%) | 1.01 (0.91, 1.12) |
| Smoked | No  Yes | | 247  92 (27%) | 118  25 (17%) | 0.64 (0.48, 0.87) |
| Pregnancy | No  Yes | | 144  28 (16%) | 78  5 (6%) | 0.37 (0.15, 0.90) |
| ^a^X denotes a suppressed cell count to control for risk of disclosure | | | | | |

eTable 6 gives (adjusted) odds ratios for participation at age 17 cross-classified according to the exposure and outcome for each analysis. Among those in the lower social classes, subjects with a record of mental illness were more likely to participate in ALSPAC at age 17 but this difference was not seen among those in the higher social classes. Therefore, the analysis carried out on participants disproportionately excludes those from the lower social classes without mental illness, resulting in a substantial overestimate of the resulting association. Similar interactions were seen for the other factors investigated, apart from breastfeeding and asthma/allergies. Those whose mother smoked during pregnancy were less likely to take part in ALSPAC at 17, but this difference was only present among those without respiratory illness; similarly, among non-smokers males were less likely to take part in ALSPAC than females but, conversely, female smokers were less likely to participate than male smokers. These interactions were not seen with respect to participation at 10 years (results not shown).

| eTable 6: Participation at age 17 years cross-classified by outcome and exposure categories | | | | | | |
| --- | --- | --- | --- | --- | --- | --- |
| Outcome | Exposure | | Outcome (before age 19) | | Adjusted OR^a^ | p-value^b^ |
|  |  |  | No | Yes | for participation |  |
| Smoked | Sex | M  F | 42/100 (42%)  53/82 (65%) | 8/18 (44%)  9/37 (24%) | 1.49 (0.49, 4.52)  0.11 (0.03, 0.38) | p=0.004 |
| Mental illness | Social class | I-IIIN  IIIM-V | 75/144 (52%)  9/37 (24%) | 22/42 (52%)  5/7 (71%) | 0.92 (0.44, 1.91)  12.08 (0.82,177.2) | p=0.04 |
| Respiratory illness | Smoking in 1st trimester | No  Yes | 60/110 (55%)  X^c^ (19%) | 53/106 (50%)  18/38 (47%) | 0.90 (0.49, 1.63)  4.64 (1.00, 21.65) | p=0.06 |
| Asthma / allergies | Breastfed | No  Yes | 9/36 (25%)  50/91 (55%) | 17/41 (41%)  64/117 (55%) | 1.44 (0.45, 4.64)  1.02 (0.56, 1.87) | p=0.3 |
| ^a^ Subgroup-specific OR for recent participation comparing those who experienced the outcome to those who didn’t, adjusted for parity, mother’s education and, sex, breastfeeding and smoking in the first trimester when these were not the exposure variables.  ^b^Interaction test  ^c^X denotes a supressed cell count to control for risk of disclosure. | | | | | | |

Discussion

If an outcome variable is missing not at random (MNAR) – being missing depends on the (unobserved) value of the outcome itself, even after taking account of other (observed) factors predictive of non-response – then both a complete case analysis, which includes only individuals with complete data, and a standard implementation of multiple imputation will generally produce biased results. However, if the data are being used to estimate an odds ratio, this will only be biased when, in addition to the outcome being MNAR, missingness in the outcome also depends on the exposure variable.^3^ Further, if there is an interaction present between the exposure and the outcome in terms of the probability of response then the bias in the odds ratio will be increased. In this study we have shown that female smokers were much less likely than female non-smokers to participate in ALSPAC in late adolescence; in contrast, the probability of taking part during this phase among males did not vary according to their smoking status. Thus, the estimated odds ratio for smoking comparing females to males was substantially different among those who had participated at 17 and those who had not. Similarly, adolescents with a low family occupational social class were much more likely to participate in ALSPAC if they had ever had a diagnosis of mental illness, but among adolescents with a high social class the probability of participating was very similar among those with and without a mental illness.

Other studies that have looked at the effect of loss to follow up on exposure-outcome associations have generally shown that the resulting bias is relatively small.^4-13^ However, in the Danish National Birth Cohort^9^ the ROR for the association between maternal smoking and ADHD was 1.33; other RORs were all close to unity and the authors concluded that the bias from loss to follow up may be large for behavioural factors. In their study of socio-economic inequalities, Howe et al^14^ found that the bias was greater as the proportion of non-participants increased (they considered participation at age 11 and at age 15). Our study supports this, as there was less evidence for bias when considering non-response in late childhood, which would still be primarily determined by the mother, but we found indications that there may be substantial bias due to non-response during late adolescence for some of the associations considered. Similarly, the Danish National Birth Cohort looked at outcomes determined at 7 years; as in ALSPAC, study participation at this age would be determined by the mother. Participation among teenagers is likely to be determined by a complex range of factors, including socio-demographic variables as well as their engagement in risk behaviours and the presence of a particular condition which, depending on what it is, may increase or decrease a person’s likelihood of taking part in a study.^15^

Studies often take account of missing (outcome and covariate) information by either including subjects with complete data (a complete case analysis) or by using multiple imputation to impute the missing data. The default assumption when using multiple imputation is that the data are missing at random (MAR) – that is, the probability of a particular variable being missing depends only on observed data (i.e. after conditioning on the observed data, the probability of a variable being missing does not depend on its own – unmeasured – value). In the examples presented in this paper it appears that the outcomes up to age 19 could be MAR in one exposure category but missing not at random (MNAR) – i.e. the probability of a variable being missing depends on its own value even after conditioning on observed data – in the other exposure category. In this situation, neither a complete case analysis nor a standard implementation of multiple imputation would address the bias.

One unique aspect of this study is that we were able access data on subjects who were eligible to take part in ALSPAC but had never enrolled into the study; this is because the GPRD collects data on an opt-out basis. Thus, in terms of the prevalence of different outcomes, we were able to examine recruitment bias as well as bias due to loss to follow-up. Because our exposure data came from ALSPAC and not the GPRD, it was not possible to examine recruitment bias in terms of exposure-outcome associations.

The main limitation of this study is its size. At the time of linkage, the GPRD covered around 8% of the UK population; however, coverage in the south west of England is relatively low. Thus confidence intervals for the odds ratios and RORs were wide, particularly when restricting to recent participants, since the exposures chosen were strongly predictive of non-response and therefore relatively uncommon amongst those remaining in the study. Another limitation of the study is the potential introduction of bias through the use of routine primary care data to determine the outcomes. This could be introduced through either through selection mechanisms or through measurement error. Although any individual registered in a GPRD-contributing practice will appear in the database, individuals will only receive a diagnosis of the outcomes being considered if they consult their GP. Measurement error could be a particular issue if clinical diagnosis of the outcomes varied according to the exposures. It is not possible to determine the likely impact of these sources of bias.

In conclusion, we have shown that the bias due to loss to follow-up among late adolescents in some exposure-outcome associations may be substantial, although we acknowledge that this study was relatively small and the uncertainty in our estimates of bias were consequently quite large. In this study, the interactions between exposure and outcome with respect to participation resulted in the outcomes being MNAR in one of the exposure subgroups. In such situations, standard implementations of multiple imputation will not overcome the resulting bias. Furthermore, without the benefit of having linked outcome data on non-participants, it would be impossible to predict when and where these interactions are likely to occur.

References

1. Boyd A, Golding J, Macleod J et al. Cohort Profile: The ‘Children of the 90s’ – the index offspring of the Avon Longitudinal Study of Parents and Children. *Int J Epidemiol* 2012;42(1):111-27.
2. Royal College of General Practitioners/National Society for the Prevention of Cruelty to Children) Safeguarding Children Toolkit^.^ <http://www.rcgp.org.uk/pdf/CIRC_2Safeguarding%20Children%20Young%20PeopleA%20Toolkit%20for%20General%20practice.pdf>. Accessed December 12, 2011.
3. Carpenter JR and Kenward MG. *Multiple Imputation and its Application*. Chichester, UK: Wiley; 2013.
4. Wolke D, Waylen A, Samara M et al. Selective drop-out in longitudinal studies and non-biased prediction of behaviour disorders. *Br J Psychiatry*. 2009;195(3):249-56.
5. Bildt C, Alfredsson L, Punnett L, Theobald H, Torgen M, Wikman A. Effects of drop out in a longitudinal study of musculoskeletal disorders. *Occup Environ Med.* 2001;58(3):194-9.
6. Ferrie JE, Kivimaki M, Singh-Manoux A et al. Non-response to baseline, non-response to follow-up and mortality in the Whitehall II cohort. *Int J Epidemiol* 2009;38(3):831-7.
7. Gustavson K, von Soest T, Karevold E, Roysamb E. Attrition and generalizability in longitudinal studies: findings from a 15-year population-based study and a Monte Carlo simulation study. *BMC Public Health*. 2012;12(1):918.
8. Powers J, Loxton D. The impact of attrition in an 11-year prospective longitudinal study of younger women. *Ann Epidemiol.* 2010;20(4):318-21.
9. Greene N, Greenland S, Olsen J, Nohr EA. Estimating bias from loss to follow-up in the Danish National Birth Cohort. *Epidemiology.* 2011;22(6):815-22.
10. Bjertness E, Sagatun A, Green K, Lien L, Sogaard AJ, Selmer R. Response rates and selection problems, with emphasis on mental health variables and DNA sampling, in large population-based, cross-sectional and longitudinal studies of adolescents in Norway. *BMC Public Health*. 2010;10:602.
11. Tin Tin S, Woodward A, Ameratunga S. Estimating bias from loss to follow-up in a prospective cohort study of bicycle crash injuries. *Inj Prev*. 2013; 0:1-8.
12. Osler M, Kriegbaum M, Christensen U, Lund R, Anderson AN. Loss to follow-up did not bias associations between early life factors and adult depression. *J Clin Epidemiol.* 2008;61(9):958-63.
13. Osler M, Kriegbaum M, Christensen U, Holstein B, Anderson AN. Rapid report on methodology: does loss to follow-up in a cohort study bias associations between early life factors and lifestyle-related health outcomes? *Ann Epidemiol.* 2008;18(5):422-4.
14. Howe LD, Tilling K, Galobardes B, Lawlor DA. Loss to follow-up in cohort studies: bias in estimates of socioeconomic inequalities. *Epidemiology*. 2013;24(1):1-9.
15. Galea S, Tracy M. Participation rates in epidemiologic studies. *Ann Epidemiol*. 2007;17(9):643-53.
